# Association between markers of inflammation and outcomes after hip fracture surgery: analysis of routinely collected electronic healthcare data

**DOI:** 10.1186/s12877-025-05939-0

**Published:** 2025-04-24

**Authors:** Shivam N. Kolhe, Richard Holleyman, Andrew Chaplin, Sarah Langford, Mike R. Reed, Miles D. Witham, Antony K. Sorial

**Affiliations:** 1 AGE Research Group, NIHR Newcastle Biomedical Research Centre, Translational and Clinical Research Institute, Faculty of Medical Sciences, Newcastle University, Campus for Ageing and Vitality, Newcastle upon Tyne, NE4 5PL UK; 2 NIHR Newcastle Biomedical Research Centre, Newcastle upon Tyne NHS Foundation Trust, Cumbria Northumberland Tyne and Wear NHS Foundation Trust and Newcastle University, Newcastle upon Tyne, UK; 3 Department of Trauma and Orthopaedics, Northumbria Healthcare NHS Foundation Trust, Northumbria House, Cobalt Business Park, Newcastle upon Tyne, UK; 4 Population Health Sciences Institute, Faculty of Medical Sciences, Newcastle University, Newcastle upon Tyne, UK; 5 Institute for Cell and Molecular Biosciences, Faculty of Medical Sciences, Newcastle University, Newcastle upon Tyne, UK

**Keywords:** Hip fracture, Post-operative outcomes, Inflammation, Biomarkers, Albumin, Frailty, CRP, Prognostication

## Abstract

**Background:**

Risk assessment tools such as the Nottingham Hip Fracture Score (NHFS) are crucial in guiding prognostic discussions and benchmarking in hip fracture care. These scores have scope to be improved, which may help identify higher-risk patients at admission. We investigated the role of inflammatory biomarkers, which are routinely collected at admission, in predicting post-operative outcomes following hip fracture. We subsequently combined these biomarkers with the NHFS to see if we could enhance risk prediction.

**Methods:**

We analysed data from patients admitted to a trauma unit with hip fracture between 2015 and 2020 who underwent operative management. National hip fracture database (NHFD) data, including the NHFS, were linked with admission biomarkers: albumin, C-reactive protein (CRP), neutrophil-lymphocyte ratio (NLR) and monocyte-lymphocyte ratio (MLR). Following univariate and multivariate analyses, the discrimination of the NHFS with and without each biomarker was assessed for 30-day mortality, length of stay (LOS), and failure to return home at 30 days.

**Results:**

We analysed 1039 patients, 719 (69.2%) were female and the mean age was 82.5 years (SD 8.1, range 60–104). In multivariate analysis, higher CRP was associated with higher 30-day mortality (odds ratio (OR) 1.23, 95%, confidence interval (CI) 1.04–1.44, *p* = 0.013); higher albumin was associated with lower 30-day mortality (OR 0.86, 95%CI 0.81–0.91, *p* < 0.001). Independent predictors of not returning home at 30 days included albumin (OR 0.94, 95% CI 0.91–0.98) and NLR (OR 1.44, 95% CI 1.14–1.81). NLR and MLR were significantly associated with prolonged LOS but not 30-day mortality. A composite variable of NHFS and albumin had better discrimination for 30-day mortality than NHFS alone (c-statistics 0.74, 95% CI 0.68–0.80 vs. 0.68, 95% CI 0.62–0.75, respectively). CRP, NLR and MLR did not improve discrimination for any outcome when added to NHFS.

**Conclusions:**

Albumin, but not other markers of inflammation, enhances risk prediction after hip fracture when added to the NHFS. Routine recording of albumin at admission may have a future role in an enhanced risk scoring system for prognostication in hip fracture surgery.

**Supplementary Information:**

The online version contains supplementary material available at 10.1186/s12877-025-05939-0.

## Background

Approximately 75,000 individuals each year sustain a hip fracture in the UK with associated increased mortality, morbidity and a reduction in quality of life [1]. Current management involves complex multidisciplinary care to meet the needs of this vulnerable and frail patient population, often with prolonged hospital stays and complex discharge planning. Direct costs of hip fractures to the NHS are an estimated £2 billion annually [2–4]. With an ageing population and more older people surviving with frailty and multimorbidity, the incidence of hip fractures is expected to rise globally [5].

Despite national audit initiatives such as the National Hip Fracture Database (NHFD), 30-day mortality rates remain high [6]. The Nottingham Hip Fracture Score (NHFS) is a widely validated, pre-operative risk score derived from well-known indicators of adverse surgical outcomes including older age, male sex, admission haemoglobin (≤ 10 g/dL), living in an institution, multiple comorbidities, previous malignancy, and Abbreviated Mental Test Score (AMTS ≤ 6) [7]. It is mandated as part of NHFD data collection and was originally developed to predict 30-day mortality, it also demonstrates moderate discriminant ability for 30-day mortality but is poor at predicting length of stay (LOS) [8]. There is therefore scope for enhancing prediction of a wider range of clinically relevant outcomes including discharge destination (a proxy for physical function and independence), LOS, and complications. This could lead to the identification of high-risk hip fracture patients at admission, aiding patient-specific care decisions, and informing discussions on prognosis, resuscitation, and discharge planning. Improved prediction of clinical outcomes within the routinely performed NHFS can be used to supplement clinical judgement for clinicians when facilitating case-mix adjustment and benchmarking at an organisational level.

Measures of inflammation may provide prognostic information that can enhance the risk-predictive capabilities of scores such as the NHFS. Chronic low-grade inflammation is thought to be a contributor to both sarcopenia (the age-related loss of muscle strength and mass) [9], and frailty (the loss of homeostatic reserve that renders individuals more vulnerable to a stressor) [10]. Acute inflammation, caused either by the fracture itself or by acute intercurrent illness, may act as a marker of allostatic load [11], again identifying a group of patients at high risk of peri-operative decompensation because of physiological stress and decreased reserve. Several studies have reported various inflammatory biomarkers, including serum albumin (a negative acute-phase protein), C-reactive protein (CRP), neutrophil-lymphocyte ratio (NLR), and monocyte-lymphocyte ratio (MLR), as being associated with adverse outcomes following hip fracture surgery [12–17]. Low albumin and elevated CRP levels have been linked to increased post-operative mortality and complications after hip fracture [13, 17–22]. NLR has recently gained prominence as a useful prognostic marker in various medical and surgical specialities [23, 24]. It is sensitive to acute inflammation and physiological stress, reflecting both innate and adaptive immune responses making it superior to absolute white cell or individual subtype counts, particularly in acute settings [25]. NLR and MLR have both been associated with post-operative mortality in hip fracture patients [15, 26]. However, there is limited evidence regarding the utility of these routinely collected and relatively inexpensive inflammatory biomarkers in enhancing the predictive ability of existing prognostic tools like the NHFS.

The primary objective of this study was to assess the association between routinely collected admission inflammatory biomarkers and post-operative outcomes following hip fracture. We also assessed whether combining baseline inflammation status with the NHFS improved the discriminant ability of the NHFS, and whether this approach could therefore be used to enhance risk prediction at admission.

## Methods

### Study design and data source

We analysed prospectively collected data on consecutive patients with hip fractures admitted to a large-volume trauma centre (Northumbria Healthcare NHS Foundation Trust) over a 5-year period, from 1st January 2015 to 29th February 2020. Baseline NHFD data and post-operative outcomes were prospectively collected by trained specialist nurses during admission. NHFD data were merged with routinely collected admission blood test results. All data were managed following Caldicott principles, ensuring confidentiality and privacy. The analysis was deemed not to require evaluation by an NHS research ethics committee since it used anonymised routinely collected data. The sources and types of data collected are summarised in Supplementary Table 1.

### Blood markers of inflammation

Results of routine biochemistry and haematology blood tests were extracted from the hospital’s Integrated Clinical Environment (ICE). To investigate the association between blood markers of inflammation and post-operative outcomes, we selected a series of inflammatory biomarkers that were likely to be routinely measured on all or most patients admitted with a hip fracture. These biomarkers included serum albumin (a negative acute-phase reactant), C-reactive protein (CRP), neutrophil-lymphocyte ratio (NLR), and monocyte-lymphocyte ratio (MLR). These measurements were chosen based on their relevance to inflammation, frailty, and association with post-operative outcomes [12–17]. We selected the first measurements of each blood test taken within 24 h of admission or pre-operatively.

### Post-operative outcomes

The primary outcome variable was mortality within 30 days of hip fracture admission. Secondary outcomes included (a) length of hospital stay (LOS), defined as the time between admission and discharge, and (b) discharge to own home, or failure to return home at 30 days.

### Exclusions

**Fig. 1:**
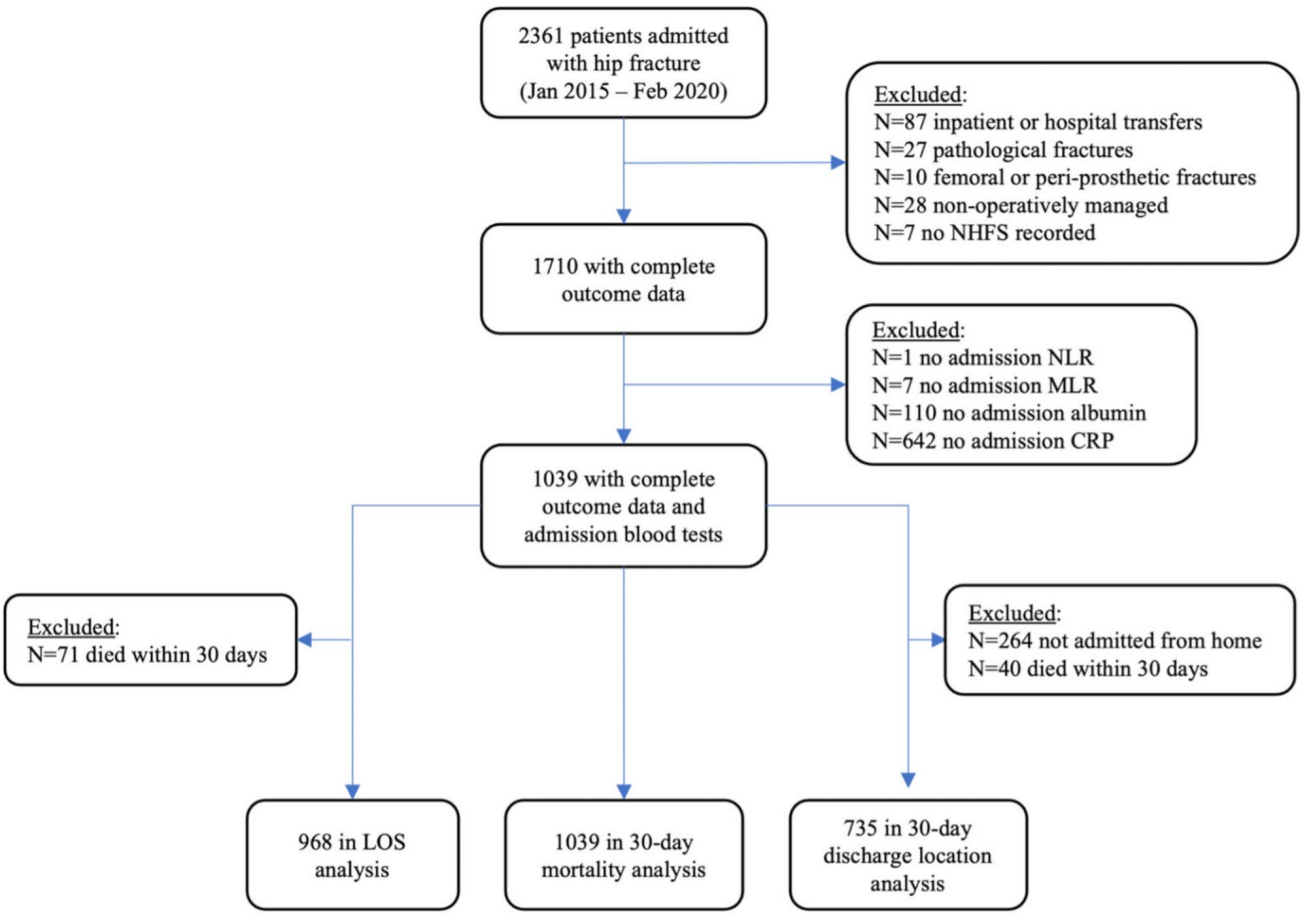
Study flowchart showing patients included in the analyses and reasons for exclusion NHFS: Nottingham Hip Fracture Score; CRP: C-reactive protein; NLR: Neutrophil-to-lymphocyte ratio; MLR: Monocyte-to-lymphocyte ratio

Patients were excluded from the analysis if they sustained a hip fracture as an inpatient, were transferred from another hospital, had pathological fractures, had a hip fracture that was non-operatively managed, or did not have a recorded NHFS on admission. The univariate and multivariate regression analyses for post-operative location at 30 days included only patients who were admitted from their homes or sheltered housing. For post-operative location and LOS analyses, patients who had died within 30 days of surgery were excluded. Additionally, patients missing any of the investigated biomarkers within 24 h of admission were excluded from all analyses. We have outlined patient exclusions from our cohort in Fig. 1, while Supplementary Table 2 compares baseline characteristics and outcomes between the full cohort (*n* = 1,710) and the analysed cohort (*n* = 1,039).

### Statistical analyses

Continuous data are presented as the mean (95% confidence interval) or the median (interquartile range) as appropriate, and categorical data are presented as percentages. Outcomes were collapsed into dichotomous variables (dead or alive at 30 days, length of stay < 28 or ≥ 28 days, discharge home or failure to return home). We performed an analysis of variance (ANOVA) test for parametric data and Mann-Whitney U test for non-parametric continuous variables to analyse differences in inflammatory biomarkers between categories of each outcome. We performed binary logistic regression analyses for each dichotomised outcome, including each combination of an inflammatory biomarker and the NHFS as independent variables.

Each biomarker was combined separately with the NHFS given the expected collinearity between measures of inflammation. The discriminant ability of each NHFS and biomarker combination was then evaluated by generating predicted probabilities from the logistic regression analyses and using these to construct receiver operator characteristic (ROC) curves. The c-statistic with a 95% confidence interval was derived for each combination and for each dichotomised outcome. C-statistics were described by convention as good (> 0.70), moderate (0.60–0.70), or poor (< 0.60) discrimination. Statistical analyses were performed using SPSS v27 (IBM, New York, USA) and certain figures generated using Prism 10.2.1 (GraphPad Software). A two-sided *p*-value < 0.05 was considered statistically significant for all analyses.

## Results

A total of 1039 patients who were admitted between 1st January 2015 and 30th February 2020, and who had complete outcome data and a complete panel of inflammatory biomarkers including albumin, CRP, NLR and MLR on admission, were included in the analyses for each post-operative outcome (Fig. 1). Baseline details for the cohort analysed are shown in Table 1. 69 patients (6.6%) of patients died within 30 days of surgery. Among patients analysed for length of stay and post-operative discharge location, 269 (27.8%) had a LOS ≥ 28 days, and 283 (38.5%) did not return to their own home within 30 days.

**Table 1:** Baseline characteristics and postoperative outcomes for all included patients

Variables	30-day mortality analysis (*n* = 1039)	LOS analysis* (*n* = 968)	30-day discharge location analysis* (*n* = 735)
Mean age (years) (SD)	82.5 (8.10)	82.2 (8.10)	81.2 (7.91)
Female sex (%)	719 (69.2)	668 (69.0)	524 (67.6)
Mean NHFS (SD)	4.99 (1.50)	4.93 (1.49)	4.50 (1.31)
***Admission blood tests***			
Mean Albumin (g/L) (SD)	38.4 (4.14)	38.7 (3.98)	39.1 (4.05)
Median C-reactive protein (mg/L) (IQR)	7.0 (2.0–28.0)	7.0 (2.0–27.0)	6.0 (2.0–24.0)
Median Neutrophil count (x10^9^/L) (IQR)	9.4 (7.0–12.1)	9.4 (7.0–12.0)	9.6 (7.1–12.2)
Median Lymphocyte count (x10^9^/L) (IQR)	1.0 (0.7–1.4)	1.0 (0.7–1.4)	1.0 (0.7–1.4)
Median NLR (IQR)	9.0 (5.8–14.0)	9.0 (5.7–13.9)	9.3 (6.1–14.3)
Median Monocyte count (x10^9^/L) (IQR)	0.7 (0.5–0.9)	0.7 (0.5–0.9)	0.7 (0.5–0.9)
Median MLR (IQR)	0.7 (0.5–1.0)	0.7 (0.5–1.0)	0.7 (0.5–1.0)
***Pre-fracture residential status*** (%)			
Own home/sheltered housing	775 (74.6)	733 (75.7)	735 (100)
Residential care	217 (20.9)	191 (19.7)	-
Nursing care	41 (3.9)	38 (3.9)	-
Other hospital site / trust	6 (0.6)	6 (0.6)	-
***Study outcomes***			
30-day mortality (%)	69 (6.6)	-	-
Median length of stay (IQR)	18.0 (10.0–28.0)	18.0 (10.0–28.0)	19.0 (11.0–31.0)
Length of stay ≥ 28 days (%)	273 (26.3)	269 (27.8)	223 (30.3)
Did not return home within 30 days (%)	587 (56.5)	516 (53.3)	283 (38.5)

NHFS: Nottingham Hip Fracture Score; CRP: C-reactive protein; NLR: Neutrophil-to-lymphocyte ratio; MLR: Monocyte-to-lymphocyte ratio. IQR: interquartile range; SD: standard deviation. *Analysis of hospital LOS and postoperative discharge destination at 30 days excluded any patients who died within this period. *Analysis of postoperative discharge destination at 30 days only included patients admitted from their own homes or sheltered housing

### Univariate associations

Serum albumin was lower, and NHFS was higher in those who died within 30 days, had a LOS ≥ 28 days, or did not return to their own home. Admission NLR and MLR was higher in those with a prolonged LOS ≥ 28 days or did not return home. Serum CRP was higher in those who died within 30 days or did not return to their own home. Full data are presented in Table 2.

**Table 2:** Univariate comparisons of clinical parameters, including admission NHFS and selected inflammatory biomarkers, with postoperative outcomes

	Alive at 30 days *N* = 970	Died within 30 days *N* = 69	*p*	LOS < 28 days* *N* = 699	LOS ≥ 28 days* *N* = 269	*p*	Returned home within 30 days* *N* = 452	Did not return home within 30 days* *N* = 283	*p*
Mean NHFS (SD)	4.9 (1.5)	5.9 (1.4)	**< 0.001** ^**a**^	4.8 (1.5)	5.2 (1.4)	**< 0.001** ^**a**^	4.2 (1.3)	5.0 (1.2)	**< 0.001** ^**a**^
Mean albumin, g/L (SD)	39 (4)	35 (5)	**< 0.001** ^**a**^	39 (4)	38 (4)	**0.006** ^**a**^	40 (4)	38 (5)	**< 0.001** ^**a**^
Median CRP, mg/L (IQR)	7 (2–27)	14 (6–53)	**0.002** ^**b**^	7 (2–24)	8 (3–36)	0.087^b^	5 (2–20)	8 (3–30)	**0.036** ^**b**^
Median NLR (IQR)	9.0 (5.7–13.9)	10.3 (7.1–15.8)	0.086^b^	8.8 (5.5–13.2)	9.6 (6.4–15.9)	**0.001** ^**b**^	9.09 (5.7–13.4)	9.9 (6.5–16.3)	**0.002** ^**b**^
Median MLR (IQR)	0.7 (0.5–1.0)	0.8 (0.5–1.2)	0.055^b^	0.6 (0.5–1.0)	0.7 (0.5–1.1)	**0.026** ^**b**^	0.7 (0.5–1.0)	0.7 (0.5–1.1)	**0.037** ^**b**^

NHFS: Nottingham Hip Fracture Score; CRP: C-reactive protein; NLR: Neutrophil-to-lymphocyte ratio; MLR: Monocyte-to-lymphocyte ratio. IQR, interquartile range; SD, standard deviation. *p*-value using (a) One-way ANOVA and (b) Mann-Whitney U test. Significant results are highlighted in bold. *Analysis of hospital LOS and postoperative discharge destination at 30 days excluded any patients who died within this period. Analysis of postoperative discharge destination at 30 days only included patients admitted from their own homes or sheltered housing

Additional analyses of further inflammatory biomarkers (Supplementary Table 3) show that monocyte counts alone were not significantly associated with any outcomes, but neutrophil and lymphocyte counts were significantly lower in patients with adverse outcomes including prolonged LOS and failure to return home, but not for 30-day mortality. Including patients who died within 30 days in the analyses for prolonged LOS or failure to return home by 30 days, did not change the results substantially, except for CRP which was significantly higher in patients with a prolonged LOS or who died (Supplementary Tables 4 and 5).

### Binary logistic regression analyses

**Fig. 2:**
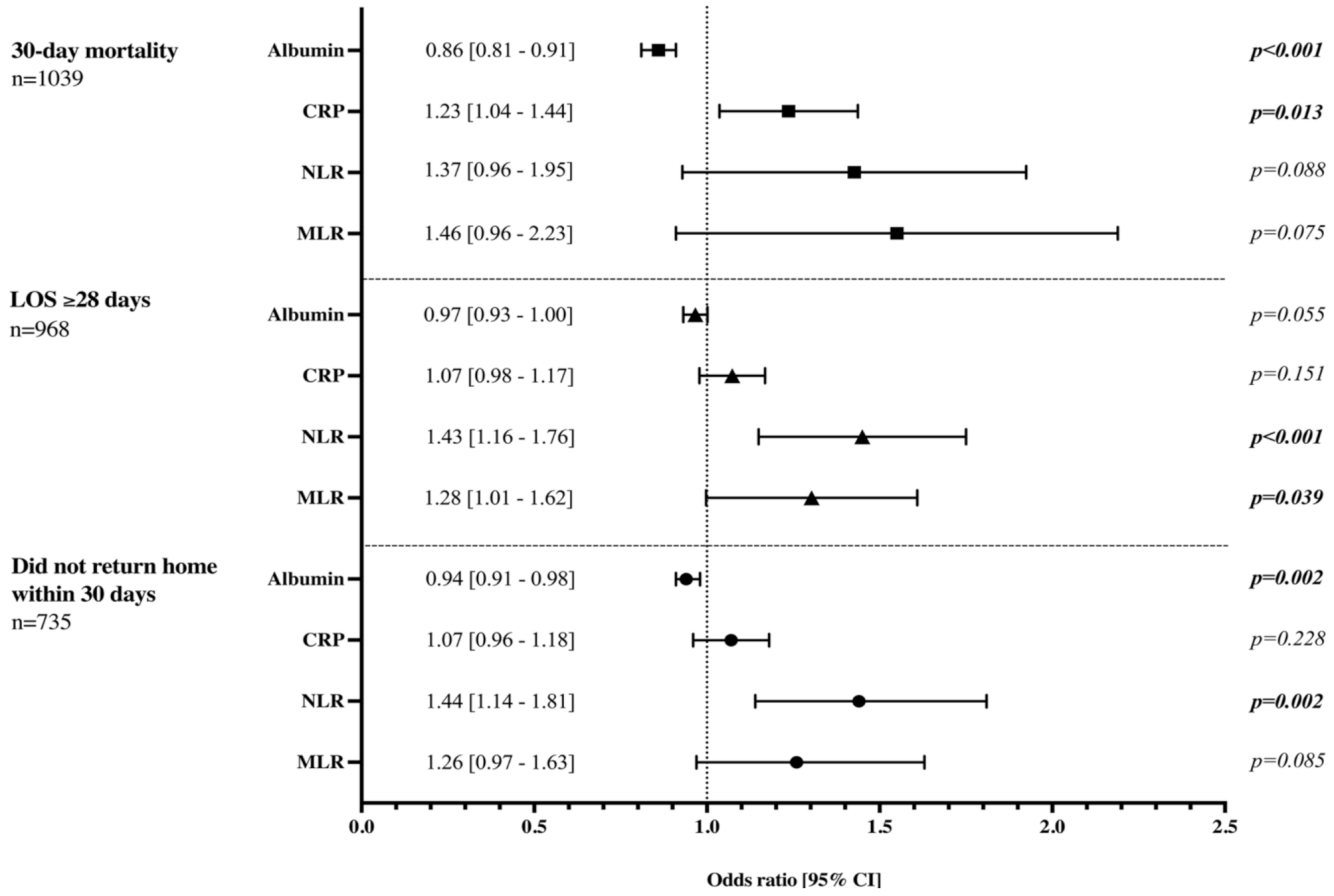
Binary logistic regression results for each biomarker, adjusted for Nottingham Hip Fracture Score (NHFS), on post-operative outcomes LOS: Length of stay; CRP: C-reactive protein; NLR: Neutrophil-to-lymphocyte ratio; MLR: Monocyte-to-lymphocyte ratio Each biomarker is analysed separately with the NHFS as a composite variable for each study outcome, with each point and error bar representing independent regression models. Analysis of post-operative discharge destination at 30 days only included patients admitted from their own homes or sheltered housing. Analysis of hospital LOS and post-operative discharge destination at 30 days excluded any patients who died within this period

In binary logistic regression analyses, adjusting for NHFS, higher CRP was associated with an increased mortality at 30 days (OR 1.23, 95% CI 1.04–1.44, *p* = 0.013), while higher admission albumin was independently associated with reduced mortality (OR 0.86, 95% CI 0.81–0.91, *p* < 0.001) (Fig. 2). Admission NLR and MLR were independently associated with a prolonged LOS (OR 1.43, 95% CI 1.16–1.76, *p* < 0.001 and OR 1.28, CI 1.01–1.62, *p* = 0.039). Higher NLR was associated with increased risk of not returning home (OR 1.44, 95% CI 1.14–1.81, *p* = 0.002), whereas higher admission albumin was associated with a lower risk of not returning home at 30 days (OR 0.94, 95% CI 0.91–0.98, *p* = 0.002).

### Discrimination analysis

**Fig. 3:**
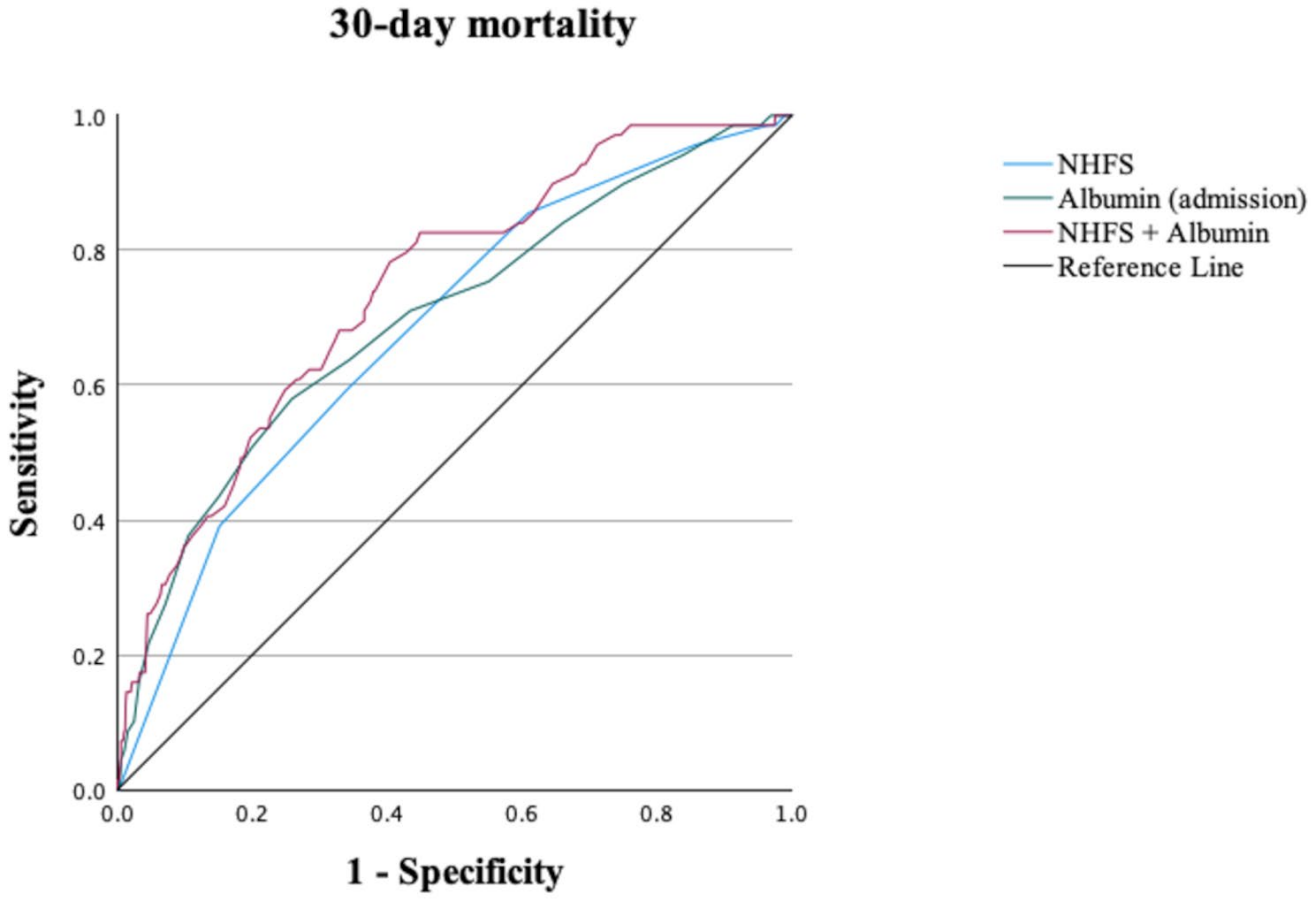
Receiver-operator characteristic (ROC) analysis of baseline NHFS, admission albumin and combination (NHFS + albumin) for 30-day mortality following hip fracture surgery NHFS: Nottingham Hip Fracture Score

The NHFS alone demonstrated moderate discrimination for predicting 30-day mortality in this cohort, with a c-statistic of 0.68 (95% CI 0.62–0.75). The NHFS performed significantly better than admission CRP, NLR, and MLR for 30-day mortality (c-statistics of 0.61 [95% CI 0.55–0.68], 0.56 [95% CI 0.50–0.63] and 0.57 [95% CI 0.50–0.64] respectively). However, admission albumin performed equally as well as the NHFS in discriminating 30-day mortality with a c-statistic of 0.70 (0.62–0.75) and when combined with the NHFS further enhanced the discriminant ability with a c-statistic of 0.74 (0.68–0.80) (Table 3; Fig. 3).

**Table 3:** C-statistics with 95% confidence intervals (CI) demonstrating the discriminant ability of each combination of NHFS and inflammatory biomarker for each postoperative outcome

	NHFS	NHFS + Albumin	NHFS + CRP	NHFS + NLR	NHFS + MLR
Mortality at 30 days (*n* = 1039)	0.68 (0.62–0.75)	0.74 (0.68–0.80)	0.70 (0.64–0.76)	0.70 (0.62–0.75)	0.70 (0.64–0.76)
Length of stay ≥ 28 days (*n* = 968)*	0.58 (0.54–0.61)	0.59 (0.55–0.63)	0.58 (0.55–0.62)	0.60 (0.57–0.64)	0.59 (0.55–0.63)
Did not return home at 30 days (*n* = 735)*	0.66 (0.62–0.70)	0.68 (0.64–0.72)	0.67 (0.63–0.71)	0.68 (0.64–0.72)	0.67 (0.63–0.71)

NHFS: Nottingham Hip Fracture Score; CRP: C-reactive protein; NLR: Neutrophil-to-lymphocyte ratio; MLR: Monocyte-to-lymphocyte ratio. *Analysis of postoperative discharge destination at 30 days only included patients admitted from their own homes or sheltered housing. Analysis of hospital LOS and postoperative discharge destination at 30 days excluded any patients who died within this period

The NHFS demonstrated poor discrimination for hospital LOS ≥ 28 days, but moderate discrimination at predicting those who did not return to their own home at 30 days. Adding admission CRP, NLR, and MLR to NHFS did not improve the discriminant ability for any of these outcomes.

## Discussion

Using prospectively collected data over a five-year period, we identify that admission albumin is an independent predictor of 30-day mortality following hip fracture. The addition of admission albumin to the NHFS enhanced the discriminant ability of NHFS for 30-day mortality but not for prolonged LOS or failure to return home at 30 days. Other inflammatory biomarkers (CRP, NLR and MLR) were either not independently associated with post-operative outcomes or did not add discriminant ability when combined with the gold-standard prognostic score (NHFS).

The NHFS is a widely used pre-operative scoring system in hip fracture surgery and has been independently validated in multiple centres worldwide [27]. Compared with other prognostic scores, such as the American Society of Anesthesiologists (ASA) grade or AMTS, the NHFS consistently demonstrates moderate yet consistent performance in predicting short-term mortality in diverse patient populations [8, 28]. Its widespread adoption, clinical relevance, and ease of use make it a suitable benchmark for evaluating the additional prognostic value of inflammatory biomarkers in this study. Consequently, our modest improvement in discriminatory ability of the NHFS with addition of albumin should be viewed in context of the existing strength of the NHFS. Our findings suggest that incorporating admission albumin levels into the NHFS could enhance its predictive accuracy for 30-day mortality after hip fracture surgery. Clinically, this could aid in identifying high-risk patients who may benefit from targeted interventions, such as nutritional support or more intensive post-operative monitoring. Future studies might focus on incorporation of albumin alongside novel prognostic tools for adverse post-operative outcomes, such as hand grip strength, into an updated NHFS [29, 30]. Collection of larger multi-centre data could both validate our findings and determine optimal cut-off points for hypoalbuminaemia.

Our study demonstrated that albumin independently predicted 30-day mortality, while other inflammatory markers added no significant value. Our findings are consistent with several previous studies evaluating hypoalbuminemia as an independent predictor of increased mortality after hip fracture [12, 17, 31]. As a negative acute-phase reactant, albumin production is switched off by the liver in response to acute or chronic inflammatory stimuli, enabling protein production to be diverted to proteins required for the inflammatory response and accelerating albumin catabolism [32, 33]. In addition to inflammation, albumin is a powerful integrative biomarker of overall health deterioration across multiple physiological systems, including malnutrition, liver and gastrointestinal disease, and is associated with frailty [34, 35]. We posit the role of CRP, NLR and MLR as transient markers of acute inflammation may not have captured underlying physiological status and may simply be reflective of the acute phase response to fracture.

Two meta-analyses by Laulund et al. and Li et al., have highlighted the potential prognostic value of admission serum albumin as an independent predictor of inpatient mortality, which is concordant with our report [36, 37]. Other previous studies have reported that low admission albumin is an independent predictor of increased hospital LOS and complications, even after accounting for various baseline characteristics and comorbidities [17, 18]. The authors of these studies attribute this association to an increased complication rate, subsequently affecting LOS. Furthermore, hypoalbuminaemia has been associated with a slower functional recovery post-operatively, contributing to extended hospital stays [38].

Previously it has been reported that NLR but not MLR is a significant predictor of 1-year mortality in patients aged over 60 years with hip fractures [39]. Our study utilised NHFD 30-day mortality data, as we did not routinely collect 1-year mortality, therefore, there may be a delayed predictive value not being captured in our dataset, a limitation of this study. Of note, this study was in a Turkish cohort which is likely to have a different ethnic and genetic composition. It has long been established that sex and ethnic origin should be taken into consideration when assessing white cell counts [40].

Lower albumin and higher NLR were both independently associated with prolonged LOS and with failure to return home. However, none of the inflammatory biomarkers tested improved the discriminant ability of the NHFS for either prolonged LOS or failure to return home. Both prolonged LOS and failure to return home are complex, non-linear outcomes which are likely to depend on many factors, in particular pre-fracture functional ability, nutritional status, comorbidity, organisation/logistical considerations, as well as the availability of social care [41]. Such factors, although only partially captured by the NHFS, may be more important than the impact of acute inflammation. Acute inflammation contributes to the acute reduction in muscle mass and strength seen in hospitalised patients [42, 43], and may also contribute to immune dysregulation, heightening the risks of post-operative complications [44, 45].

While our study did not examine these complications directly, previous studies have associated low serum albumin to an increase in hospital-acquired infections and a predictor of a prolonged hospital stay [46]. In our study cohort, any effect of acute inflammation on LOS and discharge location may have been minimised by the greater contribution of other factors to these outcomes. Future research could investigate the impact of peri-operative and nosocomial infections on LOS in patients with low albumin at admission. This may provide a clearer picture of the interplay between inflammation, nutritional status, and recovery trajectories in hip fracture patients.

Our study had several strengths. We used routinely collected clinical data, which is likely to be representative of current practice. Our patient cohort is largely representative of the characteristics of the national hip fracture cohort, enhancing the potential generalisability of our findings [47]. We investigated routinely measured, inexpensive inflammatory markers, and thus our findings should be easily applicable to clinical practice. To our knowledge, our study is the first to assess the discriminant ability of inflammatory biomarkers when combined with the NHFS, across a spectrum of clinically important outcomes.

Regarding limitations, as is often the case when analysing routinely collected data, not all patients had complete data. We focussed on blood results obtained within 24 hours of admission, excluding patients with blood tests outside of this timeframe. 671 patients lacked a full biochemistry panel within the specified timeframe (Fig. 1), thereby reducing the cohort available for multivariate analyses. The most omitted admission biomarker was CRP (*N* = 642), which was not routinely obtained on admission. Given not all patients had a full biochemistry panel, there could exist a selection bias in terms of who received CRP and albumin tests on admission, favouring more unwell individuals, patients with clinical presentations consistent with underlying infection or those with greater comorbidities that remained unaccounted for. We posit that these patients may have been omitted for reasons including organisational (delays, errors and lack of recorded data) and clinical judgment that a full biochemistry panel pre-operatively was unnecessary in context of fracture requiring acute operative fixation.

Our study excluded inpatient and pathological fractures, as well as non-operatively managed patients, possibly biasing the cohort towards decreased comorbidity and frailty, limiting generalisability [48, 49]. Despite our relatively large cohort size, our data was sourced from a single trust in the North East of England, therefore might lack generalisability to other regions due to factors such as deprivation and ethnic diversity which are known to effect hip fracture outcomes [50, 51]. We were not able to examine post-operative complications, such as nosocomial infection in detail in this cohort, and this is an area on which future research could focus.

## Conclusion

Inflammatory biomarkers, in particular albumin are associated with adverse post-operative outcomes in hip fracture surgery. Incorporating admission albumin into the NHFS improved its ability to predict 30-day mortality, demonstrating better discriminatory performance than either measure alone, highlighting albumin’s added value in risk stratification. Routinely assessing and incorporating albumin in admission-based risk scores could improve prognostic accuracy, thereby facilitating prognostic discussions, case-mix adjustment, and benchmarking for improved resource allocation in hip fracture care. Further research is still required to identify novel prognostic markers for other outcomes such as prolonged length of stay and failure to return home after hip fracture.

## Electronic supplementary material

Below is the link to the electronic supplementary material.

Supplementary Material 1

## Data Availability

Availability of data and materials. The data that support the findings of this study are available from Northumbria Healthcare NHS Foundation Trust, but restrictions apply to the availability of these data, which were used under license for the current study, and so are not publicly available. Data are however available from the authors upon reasonable request and with permission of Northumbria Healthcare NHS Foundation Trust. Data access requests can be made by email to antony.sorial@nhs.net.

## Abbreviations

ANOVA: Analysis of Variance
CI: Confidence Interval
CRP: C-reactive protein
ICE: Integrated Clinical Environment
IQR: Interquartile Range
LOS: Length of Stay
MLR: Monocyte-Lymphocyte Ratio
NHFD: National Hip Fracture Database
NHFS: Nottingham Hip Fracture Score
NLR: neutrophil-lymphocyte ratio
OR: Odds Ratio
ROC: Receiver Operator Characteristic

## References

[1] (RCP) RCoP. Improving Understanding: The National Hip Fracture Database Report on 2021. 2022.

[2] Judge A, Javaid MK, Leal J, Hawley S, Drew S, Sheard S, Prieto-Alhambra D, Gooberman-Hill R, Lippett J, Farmer A et al. Models of care for the delivery of secondary fracture prevention after hip fracture: a health service cost, clinical outcomes and cost-effectiveness study within a region of England. In., vol. 4. Southampton (UK): National Institute for Health Research Health Service Delieryv Res; 2016. 27748091

[3] Moppett IK, Wiles MD, Moran CG, Sahota O. The Nottingham hip fracture score as a predictor of early discharge following fractured neck of femur. Age Ageing. 2011;41(3):322–6. 22083839 10.1093/ageing/afr142

[4] Ferris H, Brent L, Sorensen J, Ahern E, Coughlan T. Discharge destination after hip fracture: findings from the Irish hip fracture database. Eur Geriatr Med. 2022;13(2):415–24. 34420192 10.1007/s41999-021-00556-7

[5] Dhanwal DK, Dennison EM, Harvey NC, Cooper C. Epidemiology of hip fracture: worldwide geographic variation. Indian J Orthop. 2011;45(1):15–22. 21221218 10.4103/0019-5413.73656 PMC3004072

[6] Neuburger J, Currie C, Wakeman R, Tsang C, Plant F, De Stavola B, Cromwell DA, van der Meulen J. The impact of a National clinician-led audit initiative on care and mortality after hip fracture in England: an external evaluation using time trends in non-audit data. Med Care. 2015;53(8):686–91. 26172938 10.1097/MLR.0000000000000383 PMC4501693

[7] Maxwell MJ, Moran CG, Moppett IK. Development and validation of a preoperative scoring system to predict 30 day mortality in patients undergoing hip fracture surgery. Br J Anaesth. 2008;101(4):511–7. 18723517 10.1093/bja/aen236

[8] Doherty WJ, Stubbs TA, Chaplin A, Reed MR, Sayer AA, Witham MD, Sorial AK. Prediction of postoperative outcomes following hip fracture surgery: independent validation and recalibration of the Nottingham hip fracture score. J Am Med Dir Assoc. 2021;22(3):663–e669662. 32893139 10.1016/j.jamda.2020.07.013

[9] Dalle S, Rossmeislova L, Koppo K. The role of inflammation in Age-Related sarcopenia. Front Physiol. 2017;8:1045. 29311975 10.3389/fphys.2017.01045 PMC5733049

[10] Clegg A, Young J, Iliffe S, Rikkert MO, Rockwood K. Frailty in elderly people. Lancet. 2013;381(9868):752–62. 23395245 10.1016/S0140-6736(12)62167-9 PMC4098658

[11] McCrory C, McLoughlin S, Layte R, NiCheallaigh C, O’Halloran AM, Barros H, Berkman LF, Bochud M. Towards a consensus definition of allostatic load: a multi-cohort, multi-system, multi-biomarker individual participant data (IPD) meta-analysis. Psychoneuroendocrinology. 2023;153:106117. 37100008 10.1016/j.psyneuen.2023.106117 PMC10620736

[12] Geleit R, Bence M, Samouel P, Craik J. Biomarkers as predictors of inpatient mortality in fractured neck of femur patients. Arch Gerontol Geriatr. 2023;111:105004. 36958149 10.1016/j.archger.2023.105004

[13] Chen BK, Liu YC, Chen CC, Chen YP, Kuo YJ, Huang SW. Correlation between C-reactive protein and postoperative mortality in patients undergoing hip fracture surgery: a meta-analysis. J Orthop Surg Res. 2023;18(1):182. 36894998 10.1186/s13018-023-03516-y PMC9996565

[14] Kim BG, Lee YK, Park HP, Sohn HM, Oh AY, Jeon YT, Koo KH. C-reactive protein is an independent predictor for 1-year mortality in elderly patients undergoing hip fracture surgery: A retrospective analysis. Med (Baltim). 2016;95(43):e5152. 10.1097/MD.0000000000005152 PMC5089100 27787371

[15] Bingol O, Ozdemir G, Kulakoglu B, Keskin OH, Korkmaz I, Kilic E. Admission neutrophil-to-lymphocyte ratio and monocyte-to-lymphocyte ratio to predict 30-day and 1-year mortality in geriatric hip fractures. Injury. 2020;51(11):2663–7. 32739153 10.1016/j.injury.2020.07.048

[16] Lizaur-Utrilla A, Gonzalez-Navarro B, Vizcaya-Moreno MF, Lopez-Prats FA. Altered seric levels of albumin, sodium and parathyroid hormone May predict early mortality following hip fracture surgery in elderly. Int Orthop. 2019;43(12):2825–9. 31256198 10.1007/s00264-019-04368-0

[17] Bohl DD, Shen MR, Hannon CP, Fillingham YA, Darrith B, Della Valle CJ. Serum albumin predicts survival and postoperative course following surgery for geriatric hip fracture. J Bone Joint Surg Am. 2017;99(24):2110–8. 29257017 10.2106/JBJS.16.01620

[18] Aldebeyan S, Nooh A, Aoude A, Weber MH, Harvey EJ. Hypoalbuminaemia-a marker of malnutrition and predictor of postoperative complications and mortality after hip fractures. Injury. 2017;48(2):436–40. 28040258 10.1016/j.injury.2016.12.016

[19] Kieffer WK, Rennie CS, Gandhe AJ. Preoperative albumin as a predictor of one-year mortality in patients with fractured neck of femur. Ann R Coll Surg Engl. 2013;95(1):26–8. 23317722 10.1308/003588413X13511609954815 PMC3964632

[20] Pimlott BJ, Jones CA, Beaupre LA, Johnston DW, Majumdar SR. Prognostic impact of pre-operative albumin on short-term mortality and complications in patients with hip fracture. Arch Gerontol Geriatr. 2011;53(1):90–4. 20684997 10.1016/j.archger.2010.06.018

[21] Sedlar M, Kvasnicka J, Krska Z, Tomankova T, Linhart A. Early and subacute inflammatory response and long-term survival after hip trauma and surgery. Arch Gerontol Geriatr. 2015;60(3):431–6. 25704919 10.1016/j.archger.2015.02.002

[22] Laggner R, Taner B, Straub J, Tiefenbock TM, Binder H, Sator T, Hajdu S, Windhager R, Bohler C. Do elevated serum C-Reactive-Protein levels excuse delayed surgery for femoral neck fractures?? Antibiot (Basel) 2023, 12(4). 10.3390/antibiotics12040738 PMC10135175 37107100

[23] Tan TP, Arekapudi A, Metha J, Prasad A, Venkatraghavan L. Neutrophil-lymphocyte ratio as predictor of mortality and morbidity in cardiovascular surgery: a systematic review. ANZ J Surg. 2015;85(6):414–9. 25781147 10.1111/ans.13036

[24] Paramanathan A, Saxena A, Morris DL. A systematic review and meta-analysis on the impact of pre-operative neutrophil lymphocyte ratio on long term outcomes after curative intent resection of solid tumours. Surg Oncol. 2014;23(1):31–9. 24378193 10.1016/j.suronc.2013.12.001

[25] de Jager CPC, van Wijk PTL, Mathoera RB, de Jongh-Leuvenink J, van der Poll T, Wever PC. Lymphocytopenia and neutrophil-lymphocyte count ratio predict bacteremia better than conventional infection markers in an emergency care unit. Crit Care. 2010;14(5):R192. 21034463 10.1186/cc9309 PMC3219299

[26] Chen YH, Chou CH, Su HH, Tsai YT, Chiang MH, Kuo YJ, Chen YP. Correlation between neutrophil-to-lymphocyte ratio and postoperative mortality in elderly patients with hip fracture: a meta-analysis. J Orthop Surg Res. 2021;16(1):681. 34794459 10.1186/s13018-021-02831-6 PMC8600895

[27] Moppett IK, Parker M, Griffiths R, Bowers T, White SM, Moran CG. Nottingham hip fracture score: longitudinal and multi-assessment. Br J Anaesth. 2012;109(4):546–50. 22728204 10.1093/bja/aes187

[28] Ferro FC, Campos MAG, Picolli TCS, De Sá Mayoral V, Soares VM, Ferreira JC, Peres LDB, Tibeau TTM, Bernardi VEC, Pereira DN et al. Performance of the Nottingham hip fracture score (NHFS) as a predictor of 30-day mortality after proximal femur fracture in an older people Brazilian cohort. Sci Rep 2025, 15(1). 10.1038/s41598-025-89869-2 PMC11830071 39955409

[29] Selakovic I, Dubljanin-Raspopovic E, Markovic-Denic L, Marusic V, Cirkovic A, Kadija M, Tomanovic-Vujadinovic S, Tulic G. Can early assessment of hand grip strength in older hip fracture patients predict functional outcome? PLoS ONE. 2019;14(8):e0213223. 31369561 10.1371/journal.pone.0213223 PMC6675283

[30] Prowse J, Jaiswal S, Gentle J, Sorial AK, Witham MD. Feasibility, acceptability and prognostic value of muscle mass and strength measurement in patients with hip fracture: a systematic review. Eur Geriatr Med. 2024;15(6):1603–14. 39614068 10.1007/s41999-024-01102-x PMC11632060

[31] Fisher A, Fisher L, Srikusalanukul W, Smith PN. Usefulness of simple biomarkers at admission as independent indicators and predictors of in-hospital mortality in older hip fracture patients. Injury. 2018;49(4):829–40. 29559183 10.1016/j.injury.2018.03.005

[32] Soeters PB, Wolfe RR, Shenkin A. Hypoalbuminemia: pathogenesis and clinical significance. JPEN J Parenter Enter Nutr. 2019;43(2):181–93. 10.1002/jpen.1451 PMC7379941 30288759

[33] Don BR, Kaysen G. Serum albumin: relationship to inflammation and nutrition. Semin Dial. 2004;17(6):432–7. 15660573 10.1111/j.0894-0959.2004.17603.x

[34] Eckart A, Struja T, Kutz A, Baumgartner A, Baumgartner T, Zurfluh S, Neeser O, Huber A, Stanga Z, Mueller B, Schuetz P. Relationship of nutritional status, inflammation, and serum albumin levels during acute illness: A prospective study. Am J Med. 2020;133(6):713–e722717. 31751531 10.1016/j.amjmed.2019.10.031

[35] Mailliez A, Guilbaud A, Puisieux F, Dauchet L, Boulanger E. Circulating biomarkers characterizing physical frailty: CRP, hemoglobin, albumin, 25OHD and free testosterone as best biomarkers. Results of a meta-analysis. Exp Gerontol. 2020;139:111014. 32599147 10.1016/j.exger.2020.111014

[36] Laulund AS, Lauritzen JB, Duus BR, Mosfeldt M, Jorgensen HL. Routine blood tests as predictors of mortality in hip fracture patients. Injury. 2012;43(7):1014–20. 22236368 10.1016/j.injury.2011.12.008

[37] Li S, Zhang J, Zheng H, Wang X, Liu Z, Sun T. Prognostic role of serum albumin, total lymphocyte count, and Mini nutritional assessment on outcomes after geriatric hip fracture surgery: A Meta-Analysis and systematic review. J Arthroplasty. 2019;34(6):1287–96. 30852065 10.1016/j.arth.2019.02.003

[38] Sim SD, Sim YE, Tay K, Howe TS, Png MA, Chang CCP, Abdullah HR, Koh JSB. Preoperative hypoalbuminemia: poor functional outcomes and quality of life after hip fracture surgery. Bone. 2021;143:115567. 32745690 10.1016/j.bone.2020.115567

[39] Tekin SB, Bozgeyik B, Mert A. Relationship between admission neutrophil/lymphocyte, thrombocyte/lymphocyte, and monocyte/lymphocyte ratios and 1-year mortality in geriatric hip fractures: triple comparison. Ulus Travma Acil Cerrahi Derg. 2022;28(11):1634–40. 36282165 10.14744/tjtes.2021.94799 PMC10277357

[40] Bain BJ. Ethnic and sex differences in the total and differential white cell count and platelet count. J Clin Pathol. 1996;49(8):664–6. 8881919 10.1136/jcp.49.8.664 PMC500612

[41] Stubbs TA, Doherty WJ, Chaplin A, Langford S, Reed MR, Sayer AA, Witham MD, Sorial AK. Using pre-fracture mobility to augment prediction of post-operative outcomes in hip fracture. Eur Geriatr Med. 2023;14(2):285–93. 37002428 10.1007/s41999-023-00767-0 PMC10113355

[42] Liu JYJ, Reijnierse EM, van Ancum JM, Verlaan S, Meskers CGM, Maier AB. Acute inflammation is associated with lower muscle strength, muscle mass and functional dependency in male hospitalised older patients. PLoS ONE. 2019;14(4):e0215097. 30986265 10.1371/journal.pone.0215097 PMC6464173

[43] Hartley P, Costello P, Fenner R, Gibbins N, Quinn E, Kuhn I, Keevil VL, Romero-Ortuno R. Change in skeletal muscle associated with unplanned hospital admissions in adult patients: A systematic review and meta-analysis. PLoS ONE. 2019;14(1):e0210186. 30608987 10.1371/journal.pone.0210186 PMC6319740

[44] Bain CR, Myles PS, Corcoran T, Dieleman JM. Postoperative systemic inflammatory dysregulation and corticosteroids: a narrative review. Anaesthesia. 2023;78(3):356–70. 36308338 10.1111/anae.15896 PMC10092416

[45] Vester H, Huber-Lang MS, Kida Q, Scola A, van Griensven M, Gebhard F, Nussler AK, Perl M. The immune response after fracture trauma is different in old compared to young patients. Immun Ageing. 2014;11(1):20. 25620994 10.1186/s12979-014-0020-x PMC4305233

[46] Delgado-Rodríguez M, Medina-Cuadros M, Gómez-Ortega A, Martínez-Gallego G, Mariscal-Ortiz M, Martinez-Gonzalez MA, Sillero-Arenas M. Cholesterol and serum albumin levels as predictors of cross infection, death, and length of hospital stay. Arch Surg. 2002;137(7):805–12. 12093337 10.1001/archsurg.137.7.805

[47] Holleyman RJ, Khan SK, Charlett A, Inman DS, Johansen A, Brown C, Barnard S, Fox S, Baker PN, Deehan D, et al. The impact of COVID-19 on mortality after hip fracture. Bone Joint J. 2022;104–B(10):1156–67. 36177635 10.1302/0301-620X.104B10.BJJ-2022-0082.R1

[48] Amen TB, Varady NH, Hayden BL, Chen AF. Pathologic versus native hip fractures: comparing 30-day mortality and Short-term complication profiles. J Arthroplasty. 2020;35(5):1194–9. 31987688 10.1016/j.arth.2020.01.003

[49] Singh I, Hooton K, Edwards C, Lewis B, Anwar A, Johansen A. Inpatient hip fractures: Understanding and addressing the risk of this common injury. Age Ageing. 2020;49(3):481–6. 32040192 10.1093/ageing/afz179

[50] Thorne K, Johansen A, Akbari A, Williams JG, Roberts SE. The impact of social deprivation on mortality following hip fracture in England and Wales: a record linkage study. Osteoporos Int. 2016;27(9):2727–37. 27098537 10.1007/s00198-016-3608-5 PMC4981619

[51] Sullivan KJ, Husak LE, Altebarmakian M, Brox WT, Sullivan KJ, Husak LE, Altebarmakian M, Brox WT. Demographic factors in hip fracture incidence and mortality rates in California, 2000–2011. Journal of Orthopaedic Surgery and Research 2015 11:1. 2016-01-08;11(1). 10.1186/s13018-015-0332-3 PMC4705624 26746904

