# Supplementary material for: Association between markers of inflammation and outcomes after hip fracture surgery: analysis of routinely collected electronic healthcare data
Source: BMC Geriatr. 2025 Apr 24;25:274. doi: 10.1186/s12877-025-05939-0 (PMC12023628; doi:10.1186/s12877-025-05939-0)
Supplement: Supplementary file 1 — Supplementary Material 1 [file 12877_2025_5939_MOESM1_ESM.docx]

**Supplementary information:**

**Association between markers of inflammation and outcomes after hip fracture surgery: analysis of routinely collected electronic healthcare data**

**Supplementary information table captions:**

**Supplementary Table 1.** Study variables and their data sources

**Supplementary Table 2.** Baseline characteristics and post-operative outcomes for original cohort vs. final cohort (with full bloods data)

**Supplementary Table 3.** Univariate associations of baseline NHFS and all inflammatory biomarkers with postoperative outcomes.

**Supplementary Table 4.** Association between baseline NHFS and admission inflammatory biomarkers with hospital length of stay (LOS), including patients who died within 30 days.

**Supplementary Table 5.** Association between baseline NHFS and admission inflammatory biomarkers with discharge destination at 30 days, including patients who died within 30 days.

**Supplementary Table 1.** Study variables and their data sources

| *Study Variable* | *Data sources* | |
| --- | --- | --- |
| *Demographics* | | |
| Age | Routinely collected by healthcare professionals on admission | Local Audit Data (NHFD) |
| Sex |  |  |
| Pre-fracture residential status |  |  |
| *Admission risk scores* | | |
| NHFS | Calculated by orthopaedic registrars on admission to ward | Local Audit Data (NHFD) |
| ASA Grade | Calculated by anaesthetic team at pre-operative assessment clinic |  |
| *Admission blood tests* | | |
| Neutrophils (x10^9^/L) | Sunquest ICE Hospital Systems | Separate database of haematology and biochemistry blood results for hip fracture patients |
| Lymphocytes (x10^9^/L) |  |  |
| NLR |  |  |
| Monocytes (x10^9^/L) |  |  |
| MLR |  |  |
| CRP (mg/L) |  |  |
| Albumin (g/L) |  |  |
| *Operative characteristics* | | |
| Date of surgery | Routinely documented by surgeons at time of operation. | Local Audit Data (NHFD) |
| *Post-operative outcomes* | | |
| Hospital length of stay | Calculated from admission date to discharge date | Local Audit Data (NHFD) |
| Residence at 30- and 120-days | Collected by specialist NHFD nurses via telephone follow-up or documentation by medical professionals on ward. | Local Audit Data (NHFD) |
| Mortality at 30- and 120-days |  |  |

NHFD: National Hip Fracture Database; NHFS: Nottingham Hip Fracture Score; ASA: American Society of Anesthesiologists; NLR: Neutrophil-to-lymphocyte ratio; MLR: Monocyte-to-lymphocyte ratio; CRP: C-reactive protein; ICE: Integrated Clinical Environment.

**Supplementary Table 2.** Baseline characteristics and post-operative outcomes for original cohort vs. final cohort (with full bloods data)

| Variables | Original cohort  n=1710* | Final cohort with full bloods data  n=1039 |
| --- | --- | --- |
| Mean age (years) (SD) | 82.5 (8.2) | 82.5 (8.1) |
| Female sex (%) | 1199 (70.1) | 719 (69.2) |
| Mean NHFS (SD) | 4.97 (1.50) | 4.99 (1.50) |
| *Admission blood tests* |  |  |
| Mean Albumin (g/L) (SD) | 38.5 (4.1) | 38.4 (4.1) |
| Median C-reactive protein (mg/L) (IQR) | 7.0 (2.0 – 28.0) | 7.0 (2.0 – 28.0) |
| Median Neutrophil count (x10^9^/L) (IQR) | 9.3 (7.0 – 11.8) | 9.4 (7.0 – 12.1) |
| Median Lymphocyte count (x10^9^/L) (IQR) | 1.0 (0.7 - 1.4) | 1.0 (0.7 - 1.4) |
| Median NLR (IQR) | 8.8 (5.6 – 13.8) | 9.0 (5.8 – 14.0) |
| Median Monocyte count (x10^9^/L) (IQR) | 0.7 (0.5 – 0.9) | 0.7 (0.5 – 0.9) |
| Median MLR (IQR) | 0.67 (0.46 – 1.00) | 0.7 (0.5 – 1.0) |
| *Pre-fracture residential status* (%) |  |  |
| Own home/sheltered housing | 1302 (76.1) | 775 (74.6) |
| Residential care | 336 (19.6) | 217 (20.9) |
| Nursing care | 65 (3.8) | 41 (3.9) |
| Other hospital site / trust | 7 (0.4) | 6 (0.6) |
| *Study outcomes* |  |  |
| 30-day mortality (%) | 104 (6.1) | 69 (6.6) |
| Median length of stay (IQR) | 16.0 (8.0 – 27.0) | 18.0 (10.0 – 28.0) |
| Length of stay ≥28 days (%) | 413 (24.2) | 273 (26.3) |
| Did not return home within 30 days (%) | 779 (63.0) | 587 (56.5) |

NHFS: Nottingham Hip Fracture Score; CRP: C-reactive protein; NLR: Neutrophil-to-lymphocyte ratio; MLR: Monocyte-to-lymphocyte ratio.
IQR: interquartile range; SD: standard deviation. *Albumin (n=1600); CRP (n=1068); NLR (n=1709); MLR (n=1703)

**Supplementary Table 3.** Associations of baseline NHFS and all inflammatory biomarkers with postoperative outcomes.

|  | **Alive at 30 days**  N=970 | **Died within 30 days**  N=69 | **p** | **LOS <28 days***  N=699 | **LOS ≥28 days***  N=269 | **p** | **Returned home within 30 days***  N=452 | **Did not return home at 30 days***  N=283 | **p** |
| --- | --- | --- | --- | --- | --- | --- | --- | --- | --- |
| Mean NHFS (SD) | 4.9 (1.5) | 5.9 (1.4) | **<0.001^a^** | 4.8 (1.5) | 5.2 (1.3) | **<0.001^a^** | 4.2 (1.3) | 5.0 (1.2) | **<0.001^a^** |
| Mean Albumin (g/L) (SD) | 38.7 (4.0) | 35.4 (4.8) | **<0.001^a^** | 39.0 (3.8) | 38.1 (4.3) | **0.006^a^** | 39.5 (3.7) | 38.3 (4.5) | **<0.001^a^** |
| Median CRP (mg/L) (IQR) | 7.0  (2.0 – 27.3) | 14.0  (6.0 – 52.5) | **0.002^b^** | 7.0  (2.0 – 24.0) | 8.0  3.0 – 36.0) | 0.087^b^ | 5.0 (2.00 – 20.0) | 8.00  (3.00 – 30.0) | **0.036^b^** |
| Median Neutrophil (x10^9^/L) (IQR) | 9.4  (7.0 – 12.0) | 9.5  (7.5 – 13.2) | 0.348^b^ | 9.3  (6.9 – 11.8) | 9.8 (7.4 – 12.7) | **0.025^b^** | 9.3 (7.0 – 12.0) | 10.0  (7.3 – 12.7) | **0.032^b^** |
| Median Lymphocytes (x10^9^/L) (IQR) | 1.0  (0.7 – 1.4) | 0.9 (0.7 – 1.3) | 0.154^b^ | 1.1 (0.7 – 1.5) | 1.0  (0.7 – 1.3) | **0.016^b^** | 1.0  (0.7 – 1.5) | 1.0 (0.7 – 1.3) | **0.016^b^** |
| Median NLR (IQR) | 9.0  (5.7 – 13.9) | 10.3  (7.1 – 15.8) | 0.086^b^ | 8.8 (5.5 – 13.2) | 9.6  (6.4 – 15.9) | **0.001^b^** | 9.0 (5.7 – 13.4) | 9.9  (6.5 – 16.3) | **0.002^b^** |
| Median Monocyte (x10^9^/L) (IQR) | 0.7  (0.5 – 0.9) | 0.8  (0.6 – 1.0) | 0.362^b^ | 0.7  (0.6 – 0.9) | 0.7  (0.5 – 0.9) | 0.879^b^ | 0.7  (0.6 – 0.9) | 0.7  (0.5 – 0.9) | 0.943^b^ |
| Median MLR (IQR) | 0.7  (0.5 – 1.0) | 0.8  (0.5 – 1.2) | 0.055^b^ | 0.6  (0.5 – 1.0) | 0.7  (0.5 – 1.1) | **0.026^b^** | 0.7  (0.5 – 1.0) | 0.7  (0.5 – 1.1) | **0.037^b^** |

LOS: Length of stay; NHFS: Nottingham Hip Fracture Score; CRP: C-reactive protein; NLR: Neutrophil-to-lymphocyte ratio; MLR: Monocyte-to-lymphocyte ratio. P-value using (a) One-way ANOVA and (b) Mann-Whitney U test. Significant results are highlighted in bold. *Analysis of postoperative discharge destination at 30 days only included patients admitted from their own homes or sheltered housing. Analysis of hospital LOS and postoperative discharge destination at 30 days excluded any patients who died within this period

**Supplementary Table 4.** Association between baseline NHFS and admission inflammatory biomarkers with hospital length of stay (LOS), including patients who died within 30 days.

|  | **LOS <28 days**  N=699 | **LOS ≥28 days or died**  N=340 | **p** |
| --- | --- | --- | --- |
| Mean NHFS (SD) | 4.8 (1.5) | 5.4 (1.4) | **<0.001**^a^ |
| Mean Albumin (g/L) (SD) | 39.0 (3.8) | 37.5 (4.6) | **<0.001**^a^ |
| Median CRP (mg/L) (IQR) | 7.0 (2.0 - 24.0) | 9.0 (3.0 - 42.3) | **0.005^b^** |
| Median Neutrophil (x10^9^/L) (IQR) | 9.3 (6.9 - 11.8) | 9.7 (7.4 - 12.9) | **0.016**^b^ |
| Median Lymphocytes (x10^9^/L) (IQR) | 1.1(0.7 - 1.5) | 1.0 (0.7 - 1.3) | **0.005**^b^ |
| Median NLR (IQR) | 8.8 (5.5 - 13.2) | 9.9 (6.7 - 15.8) | **<0.001**^b^ |
| Median Monocyte (x10^9^/L) (IQR) | 0.7 (0.6 - 0.9) | 0.7 (0.5 - 0.9) | 0.899^b^ |
| Median MLR (IQR) | 0.6 (0.5 - 1.0) | 0.7 (0.5 - 1.1) | **0.005**^b^ |

NHFS: Nottingham Hip Fracture Score; LOS: Length of stay; CRP: C-reactive protein, NLR: Neutrophil-to-lymphocyte ratio; MLR: Monocyte-to-lymphocyte ratio. N=1037. p-value using (a) One-way ANOVA and (b) Mann-Whitney U test. Significant results are highlighted in bold.

**Supplementary Table 5.** Association between baseline NHFS and admission inflammatory biomarkers with discharge destination at 30 days, including patients who died within 30 days.

|  | Returned to own home at 30 days  N=452 | Did not return home at  30 days or died  N=323 | p |
| --- | --- | --- | --- |
| Mean NHFS (SD) | 4.2 (1.3) | 5.0 (1.2) | **<0.001**^a^ |
| Mean Albumin (g/L) (SD) | 39.5 (3.7) | 37.9 (4.7) | **<0.001**^a^ |
| Median CRP (mg/L) (IQR) | 5.0 (2.0 – 20.0) | 8.0 (3.0 – 33.0) | **005**^b^ |
| Median Neutrophils (x10^9^/L) (IQR) | 9.3 (7.0 – 12.0) | 10.0 (7.4 – 13.0) | **0.016**^b^ |
| Median Lymphocytes (x10^9^/L) (IQR) | 1.0 (0.7 - 1.5) | 1.0 (0.7 – 1.3) | **0.004**^b^ |
| Median NLR (IQR) | 9.0 (5.7 - 13.4) | 10.3 (6.7 – 16.5) | **<0.001**^b^ |
| Median Monocytes (x10^9^/L) (IQR) | 0.7 (0.6 - 0.9) | 0.7 (0.5 – 0.9) | 0.782^b^ |
| Median MLR (IQR) | 0.7 (0.5 - 1.0) | 0.7 (0.5 – 1.1) | **0.006**^b^ |

NHFS: Nottingham Hip Fracture Score; LOS: Length of stay; CRP: C-reactive protein, NLR: Neutrophil-to-lymphocyte ratio; MLR: Monocyte-to-lymphocyte ratio. N=775. p-value using (a) One-way ANOVA and (b) Mann-Whitney U test. Significant results are highlighted in bold.
